# Sensitization to alpha‐gal as a cause of idiopathic anaphylaxis

**DOI:** 10.1002/clt2.12309

**Published:** 2023-12-06

**Authors:** Thushali Ranasinghe, Inoka Sepali Aberathna, Jeewantha Jayamali, Thashmi Nimasha, Harshani Chathurangika, Deneshan Peranantharajah, Hashini Colambage, Gathsaurie Neelika Malavige, Chandima Jeewandara

**Affiliations:** ^1^ Allergy Immunology and Cell Biology Unit Department of Immunology and Molecular Medicine University of Sri Jayewardenepura Nugegoda Sri Lanka

To the Editor,

The incidence of anaphylaxis continues to rise globally based on hospital data from Europe (Sweden), United States, Australia, Brazil and some Asian (Japan) countries.^1^, ^2^, ^3^, ^4^ However, the incidence is likely to be higher due to misdiagnosis, misclassification, and underreporting^1^ and because allergies and anaphylaxis are being neglected in developing countries due to other disease priorities.^5^ In a large proportion of cases, after extensive evaluation, a trigger cannot be identified and are classified as ‘idiopathic anaphylaxis’ (IA). As food consumption patterns, genetic background, and environmental factors can lead to differences in allergen sensitization patterns in different geographical regions, we sought to identify possible triggers of IA in Sri Lankan patients presenting to a specialized allergy clinic.

All patients referred with a history of anaphylaxis were recruited following informed consent. The patients were evaluated for possible aetiological factors and contributing cofactors for the development of anaphylaxis. Relevant skin prick tests were conducted to identify the allergen. Those in whom a possible allergen could not be identified were classified as having idiopathic anaphylaxis. Accordingly, from January to December 2021, of 200 patients with anaphylaxis screened at the clinic, 65 patients were considered to have IA. In all patients, the events that led to the episode, the foods consumed, the severity of symptoms, and treatment received were recorded. Ethical approval was obtained from the Ethics Review Committee, Faculty of Medical Sciences, University of Sri Jayewardenepura, Sri Lanka. A blood sample was obtained from all patients on which an Immuno Solid‐Phase Allergen Chip (ISAC) ImmunoCAP was performed. In patients whose ISAC was negative, a serum tryptase was done and results were within the normal range. Statistical analysis was done using GraphPad prism version 9.0.

Of the 65 patients, 42 (64.6%) were females and 49 (75.38%) were adults. Eight (12.3%) had grade 1 anaphylaxis, forty‐six (70.8%) grade 2 anaphylaxis, and eleven (16.9%) grade 3 anaphylaxis. The allergen sensitization pattern is shown in (Table 1). Thirty‐four (52.3%) patients were found to have specific IgE to alpha‐gal. The other main allergens sensitized were house dust mites, twenty (30.8%), and grass pollen, sixteen (24.6%).

**TABLE 1:** Allergen sensitization pattern among patients who presented with idiopathic anaphylaxis.

Allergen	Number of patients who had specific IgE to a particular allergen (%)	Specific IgE mean, [median and range. (ISU‐E)]
Alpha‐gal	34 (52.3%)	10.8, [1.3, (0.3–100)]
House dust mite	21 (32.3%)	
*B.tropicalis*: Mite group 5 (Blo t 5)	11 (16.9%)	24.54, [23.5, (0.3–53)]
*D. farinae*	11 (16.9%)	
Cysteine protease (Der f 1)	10 (15.4%)	13.35, [3.4, (0.4–63)]
NPC2 family (Der f 2)	4 (6.2%)	42.65, [31.95, (6.7–100)]
*D. pteronyssinus*	18 (27.7%)	
Cysteine protease (Der p 1)	11 (16.9%)	24.65, [8.7, (0.8–100)]
NPC2 family (Der p 2)	7 (10.8%)	30.52, [9.95, (0.3–100)]
Peritrophin‐like protein domain (Der p 23)	13 (6.2%)	22.55, [6.15, (0.6–100)]
Grass pollen	16 (24.6%)	
Bermuda grass: Grass group 1 (Cyn d 1)	13 (20%)	2.08, [1.4, (0.5–5.8)]
Timothy grass:	16 (24.6%)	
Berberine bridge enzyme (Phl p 4)	14 (21.5%)	2.63, [1, (0.5–12)]
Grass group 5 (Phl p 5)	1 (1.5%)	0.6
Procalcin (Phl p 7)	1 (1.5%)	0.9
Profilin (Phl p 12)	1 (1.5%)	0.3
Tree pollen	9 (13.8%)	
Plane tree: Putative invertase inhibitor (Pla a 1)	1 (1.5%)	1.5
Japanese Cedar: Pectate lyase (Cry j 1)	6 (9.2%)	0.82, [0.5, (0.3–1.8)]
Cypress: Pectate lyase (Cup a 1)	6 (9.2%)	1.3, [1.25, (0.5–2.8)]
Birch: PR‐10 Protein (Bet v 1)	1 (1.5%)	0.5
Olive pollen: Lipid transfer protein (nsLTP)	1 (1.5%)	0.6
Cow's milk	11 (16.9%)	
Transferrin (Bos d lactoferrin)	9 (13.8%)	1.43, [0.8, (0.4–3.7)]
Alpha‐lactalbumin (Bos d 4)	2 (3.1%)	41.35, [41.35, (9.7–73)]
Beta‐lactoglobulin (Bos d 5)	2 (3.1%)	15.5, [15.5, (10–21)]
Casein (Bos d 8)	2 (3.1%)	69.5, [69.5, (45–94)]
Cross‐reactive components: Serum albumin (Bos d 6)	2 (3.1%)	52.85, [52.85, (5.7–100)]
Cross‐reactive carbohydrate determinants (MUXF3)	8 (12.3%)	0.74, [0.55, (0.4–2.2)]
Tropomyosin	7 (10.8%)	
Anisakis (Ani s 3)	6 (9.2%)	28.55, [28.5, (0.4–2.2)]
Cockroach (Bla g 7)	7 (10.8%)	20.29, [24, (0.4–41)]
*D. pteronyssinus* (Der p 10)	6 (9.2%)	24.58, [27.5, (4.5–46)]
Shrimp (Pen m 1)	6 (9.2%)	25.11, [27, (1–43)]
Animal: Cat ‐ Fel d 1 (Uteroglobin)	4 (6.15%)	5.95, [6.55, (0.9–9.8)]
Shrimp	3 (4.62%)	
Arginine kinase (Pen m 2)	2 (3.1%)	4.15, [4.15, (1–7.3)]
Sarcoplasmic calcium‐binding protein (Pen m 4)	1 (1.5%)	43
Wheat—*Triticum aestivum*	3 (4.62%)	
Omega‐5 gliadin (Tri a 19.0101)	2 (3.1%)	0.85, [0.85, (0.8–0.9)]
Lipid transfer protein (nsLTP) Tri a 14	1 (1.5%)	0.8
*Aspergillus fumigatus*	3 (4.62%)	
Mitogillin family (Asp f 1)	2 (3.1%)	1.05, [1.05, (0.8–1.3)]
Peroxisomal protein (Asp f 3)	1 (1.5%)	1
Soybean	2 (3.08%)	
Storage protein, Beta‐conglycinin (Gly m 5)	1 (1.5%)	1
Storage protein, Glycinin (Gly m 6)	1 (1.5%)	0.4
Storage mite: *L. destructor* ‐ NPC2 family (Lep d 2)	2 (3.08%)	1.45, [1.45, (0.8–2.1)]
Cashew‐nut: Storage protein, 11S globulin (Ana o 2)	1 (1.54%)	0.4
Weed pollen—Saltwort: Pectin methylesterase (Sal k 1)	1 (1.54%)	0.3

*Note*: All results indicated in this table were positive responses from the thermo‐fisher ISAC ImmunoCAP Test.

Allergy to Galactose‐α‐1,3‐galactose (α‐gal) has been shown as an important cause of anaphylaxis among those in whom a cause is unidentifiable.^6^ Of the patients sensitized to alpha‐gal, fourteen (41.2%) were male and twenty‐two (64.7%) were adults. Sixteen (47.1%) did not have detectable IgE to other allergens included in the ISAC ImmunoCAP. Twelve (35.3%) had consumed mammalian meat prior to developing anaphylaxis, and therefore, alpha‐gal allergy is most likely to be the trigger in these patients. Fourteen had consumed milk products, and two had consumed gelatine products prior to the reaction. In eight patients, the trigger was unknown or unrelated to alpha‐gal. 16 of 34 patients who were sensitized had never consumed mammalian meat, as its consumption is less among many ethnic groups in Sri Lanka. Therefore, although they did have specific IgE to alpha‐gal possibly following a tick bite, it is unclear if the presence of alpha‐gal specific antibodies played a role in causing anaphylaxis in these patients.

Alpha‐gal allergy typically occurs 3–6 h after the ingestion of mammalian meat and has been shown to occur due to IgE antibodies specific to the carbohydrate epitope found in mammalian meat.^7^ Patients reported symptoms between 0.5 and 6 h since ingestion of food. When considering clinical features, all (34) alpha gal patients reported an average of 2 episodes of anaphylaxis and the others (31) reported an average of 3 episodes at the time of presentation. Urticaria and itching were the most common symptoms in both groups. Difficulty in breathing, swelling of the lips, and syncope were significantly higher (*p* < 0.05) in those who were not sensitized to alpha‐gal. In contrast, diarrhea and abdominal pain were more common in those who were sensitized to alpha‐gal, although this was not significant (*p* > 0.05).

Eleven (16.9%) of all patients with IA were sensitized to cow's milk, and they were also sensitized to alpha‐gal. Interestingly, 14 of 34 patients who were found to be sensitized to alpha‐gal had consumed milk products and not mammalian meat prior to the reaction. Three of these patients had consumed fermented buffalo milk and not cow's milk. It has been shown in different studies that individuals who are sensitized to alpha‐gal react to different types of dairy products, with 70%–90% of individuals with alpha‐gal allergy reacting to milk products.^8^ It was shown that although patients with alpha‐gal allergy did not react to the main allergens in cow's milk, they reacted to bovine‐γ‐globulin, lactoferrin, and lactoperoxidase.^8^ Therefore, it is possible that in the 14 patients with alpha‐gal allergy who developed anaphylaxis following the ingestion of milk was due to sensitization to these components in milk. Ten of the fourteen were positive for major cow allergens according to the ISAC Test.

Two patients developed anaphylaxis following the consumption of gelatine‐containing products and were found to have specific antibodies to alpha‐gal. Alpha‐gal has been reported in gelatine‐containing products,^7^ and its presence could have triggered anaphylaxis. Individuals without specific IgE to gelatine but to alpha‐gal have developed anaphylaxis following the administration of vaccines due to the alpha‐gal component in gelatine.^9^ Therefore, it is recommended that gelatine containing vaccines should be administered with caution in those with alpha‐gal allergy.^9^

In summary, a large proportion of patients presenting with IA were found to be sensitized to alpha‐gal, which was the likely cause of their anaphylaxis. However, further studies need to be done to ascertain the significance of alpha‐gal sensitization among the general Sri Lankan population.

## AUTHOR CONTRIBUTIONS

**Thushali Ranasinghe**: Data curation (equal); Formal analysis (equal); Investigation (equal); Methodology (equal); Project administration (equal); Writing – original draft (equal). **Inoka Sepali Aberathna**: Data curation (equal); Investigation (equal); Methodology (equal); Project administration (equal). **Jeewantha Jayamali**: Data curation (equal); Methodology (equal). **Thashmi Nimasha**: Data curation (equal); Methodology (equal). **Harshani Chathurangika**: Data curation (equal); Methodology (equal). **Deneshan Peranantharajah**: Data curation (equal); Methodology (equal). **Hashini Colambage**: Data curation (equal); Methodology (equal). **Gathsaurie Neelika Malavige**: Conceptualization (equal); Formal analysis (equal); Funding acquisition (equal); Methodology (equal); Resources (equal); Supervision (equal); Writing – original draft (equal); Writing – review & editing. **Chandima Jeewandara**: Conceptualization (equal); Formal analysis (equal); Funding acquisition (equal); Methodology (equal); Project administration (equal); Resources (equal); Supervision (equal); Writing – original draft (equal); Writing – review & editing (equal).

## CONFLICT OF INTEREST STATEMENT

None of the authors have any conflicts of interest in relation to this manuscript.

## FUNDING INFORMATION

Allergy, Immunology and Cell Biology Unit, Faculty of Medical Sciences, University of Sri Jayewardenepura

## Data Availability

The data that support the findings of this study are available from the corresponding author upon reasonable request.
